# Self-Sensing Nonlinear Ultrasonic Fatigue Crack Detection under Temperature Variation †

**DOI:** 10.3390/s18082527

**Published:** 2018-08-02

**Authors:** Namgyu Kim, Keunyoung Jang, Yun-Kyu An

**Affiliations:** 1 Department of Civil and Environmental Engineering, Sejong University, 209 Neungdong-ro, Gwangjin-gu, Seoul 05006, Korea; namgyu.kim@outlook.com; 2 Department of Architectural Engineering, Sejong University, 209 Neungdong-ro, Gwangjin-gu, Seoul 05006, Korea; jj99137@sju.ac.kr

**Keywords:** fatigue crack detection, ultrasonic nonlinearity, self-sensing, linear and nonlinear parameters, temperature variation, structural health monitoring, nondestructive evaluation

## Abstract

This paper proposes a self-sensing nonlinear ultrasonic technique for fatigue crack detection under temperature variations. Fatigue cracks are identified from linear (*α*) and nonlinear (*β*) ultrasonic parameters recorded by a self-sensing piezoelectric transducer (PZT). The self-sensing PZT scheme minimizes the data acquisition system’s inherent nonlinearity, which often prevents the identification of fatigue cracks. Also, temperature-dependent false alarms are prevented based on the different behaviors of *α* and *β*. The proposed technique was numerically pre-validated with finite element method simulations to confirm the trends of *α* and *β* with changing temperature, and then was experimentally validated using an aluminum plate with an artificially induced fatigue crack. These validation tests reveal that fatigue cracks can be detected successfully in realistic conditions of unpredictable temperature and that positive false alarms of 0.12% occur.

## 1. Introduction

Failure of steel structures can have catastrophic consequences. Bridge collapse [1] and liquefied petroleum gas (LPG) tank explosion [2] are typical accidents associated with structural damage in steel members. Early detection of damage to steel components is therefore vital for the prevention of such accidents. Fatigue cracks, caused by repeatedly applied loads, are one of the most critical types of damage in steel structures. Fatigue cracks propagate and lead to plastic deformation over time, and are often invisible to the naked eyes, so fatigue cracks are difficult to detect before they become dangerous.

To effectively detect early-stage fatigue cracks, several nondestructive evaluation (NDE) techniques have been developed. Ultrasonic crack detection techniques have been adopted widely for NDE. Linear ultrasonic techniques have been studied which identify fatigue cracks from linear characteristics of ultrasonic signals, such as attenuation, reflection, transmission, mode conversion, and velocity change. From these characteristics, a defect can be detected simply by comparing the target’s current response to baseline data measured from a pristine target [3]. However, these linear ultrasonic characteristics are often not sensitive to incipient or closed fatigue cracks [4]. To overcome this technical limitation, various nonlinear ultrasonic techniques have been proposed. Nonlinearity in an ultrasonic signal is sensitive to clapping of closed fatigue cracks, micro-plastic deformations, and dislocations [5]. Typical approaches for observing nonlinear ultrasonic characteristics can be classified into three groups: (i) Super-harmonic, (ii) sub-harmonic, and (iii) mixed-frequency methods.

The super-harmonic method is very popular for fatigue-crack detection [6,7]. In a metallic structure, a fatigue crack will cause a nonlinear interaction with ultrasonic waves, which manifests as ultrasonic super-harmonics if the structure is excited with an ultrasonic signal of a given frequency. This method is highly sensitive to a fatigue crack’s nonlinear behavior, including micro-plastic deformation, dislocation, and clapping. However, technical difficulties arise in practice because of the inherent harmonic distortions associated with electrical sensing equipment and data acquisition systems [8]. In practice, the inherent nonlinearities coming from harmonic electrical distortions may disturb the proper observation of a fatigue crack-induced ultrasonic nonlinearity.

The sub-harmonic method tends to be more reliable than the super-harmonic approach when electrical distortion is an issue. Sub-harmonic signals are not typically generated by electrical harmonic distortion, but they are sensitively induced by a fatigue crack. However, specific conditions are required to generate sub-harmonic signals [9,10]. The sub-harmonic method requires excitation power higher than a threshold determined by the force needed to generate clapping between the crack interfaces [11]. Excitation power of this magnitude may be difficult to apply to real structures under harsh environmental and operating conditions.

The mixed-frequency method, utilizing two distinctive low and high frequency ultrasonic excitation signals together to measure the ultrasonic nonlinearity, has been widely used for fatigue crack detection. Two distinct flexural ultrasonic waveforms of different frequencies are mixed in the driving signal to produce spectral sidebands that occur as the waves interfere with each other at the fatigue crack. The applied frequencies cause the phenomenon of crack opening and closing in the phase of dilation and contraction. During the dilation phase of the low-frequency cycle, ultrasonic waves are partially decoupled by the open crack, reducing the wave amplitude as they pass through the crack. In the contraction phase of the low-frequency cycle, the closed crack does not interrupt the ultrasonic signal [12,13,14,15]. This approach is effective for detecting fatigue cracks, but some technical hurdles remain to be overcome. For example, at least two piezoelectric transducers (PZTs) are typically required to generate and measure the two distinctive ultrasonic waves. Also, environmental influences on the sidebands may cause false alarms. Furthermore, the installation location of PZT array must be carefully designed to effectively measure and analyze the nonlinear ultrasonic signals produced from the fatigue crack.

This study proposes a novel crack detection technique that exploits the super-harmonics obtained when a single self-sensing PZT drives and receives the ultrasonic signal. Since the proposed technique uses only a single PZT to generate and measure ultrasonic nonlinearities caused by a fatigue crack, the implementation problems associated with the need for sophisticated installation design and electrical distortion can be minimized [16,17]. Then, a novel fatigue-crack diagnosis algorithm is developed based on both linear (*α*) and nonlinear (*β*) signal parameters, which compensates for inherent electrical and material nonlinearities and temperature effects simultaneously. Furthermore, the simplified sensor device significantly reduces implementation and maintenance costs. To the authors’ best knowledge, no prior research has focused on temperature compensation in ultrasonic nonlinearity signals, except for preliminary test results presented by the authors at a conference [18]. The proposed technique was numerically validated using finite element method (FEM) simulations and was then experimentally validated under varying temperature conditions.

This paper is organized as follows. Section 2 explains the theoretical background and fatigue crack diagnosis algorithm. Then, Section 3 discusses the pre-validation FEM simulations that confirmed the trends in how *α* and *β* change under varying temperature conditions. In Section 4 and Section 5, the experimental validation test results and the limitations are discussed, respectively. Section 6 summarizes the paper and draws conclusions.

## 2. Theoretical Development

According to Hooke’s law, the relationship between stress (*σ*) and strain (*ε*) can be expressed in terms of nonlinearity in the elastic behavior of a material, as in Equation (1):

(1) $$\sigma =E\varepsilon \left(1+\beta \varepsilon +\delta \varepsilon^{2}+\cdots \right),$$

where E is Young’s modulus. β and δ denote the 2nd- and 3rd-order nonlinear elastic coefficients, respectively. This equation assumes that attenuation and δ make only negligible contributions to σ.

Then, the equation of motion for longitudinal and planar waves in a thin circular rod can be derived from the following nonlinear stress–strain relationship.

(2) $$\rho \frac{\partial^{2}u}{\partial t^{2}}=\frac{\partial \sigma }{\partial x},$$

where ρ is the density of the medium, x is the propagation distance, t is time, and u is the displacement.

The nonlinear wave equation for displacement u up to the 2nd-order nonlinear term is represented as follows:

(3) $$\rho \frac{\partial^{2}u}{\partial t^{2}}=E\frac{\partial^{2}u}{\partial x^{2}}+E\beta \frac{\partial u}{\partial x}\frac{\partial^{2}u}{\partial x^{2}}.$$

Perturbation theory is applied to solve Equation (3) [19]. The displacement u is assumed as follows:

(4) $$u=u_{0}+u^{\prime },$$

where $u_{0}$ represents the initially excited wave and $u^{\prime }$ represents the 1st-order perturbation solution.

The 2nd-order perturbation solution can be obtained as follows [20].

(5) $$u=u_{0}+u^{\prime }=A_{1}cos\left(kx-\omega t\right)+A_{2}sin\left(2kx-2\omega t\right),$$

where k is the wavenumber and $A_{1}$ represents the magnitude of the 1st-order harmonic wave, which is referred to as the primary-wave mode. Then, the nonlinear component $u^{\prime }$ with a magnitude of $A_{2}$, which characterizes the nonlinear attributes of the measured ultrasonic wave signal, has a nominal frequency of 2ω (double the excitation frequency). This indicates that nonlinearity in the propagation medium distorts the incident mono-frequency. $A_{2}$ is given by:

(6) $$\;A_{2}=\frac{\beta }{8}A_{1}^{2}k^{2}x,$$

where

(7) $$\beta =\frac{8}{A_{1}^{2}k^{2}x}A_{2}.$$

Once the properties of the target material, the sensor location, and the driving frequency are determined, k and x are constant. Thus, Equation (7) can be rewritten as:

(8) $$\beta =\zeta \frac{A_{2}}{A_{1}^{2}},$$

where ζ is an arbitrary constant that corresponds to *k* and *x*.

Next, the linear parameter α can be defined with the linear relationship between the arbitrary input amplitude ($A_{0}$) and $A_{1}$, because $A_{1}$ is linearly proportional to $A_{0}$.

(9) $$\alpha =\frac{A_{1}}{A_{0}}.$$

**Figure 1:**
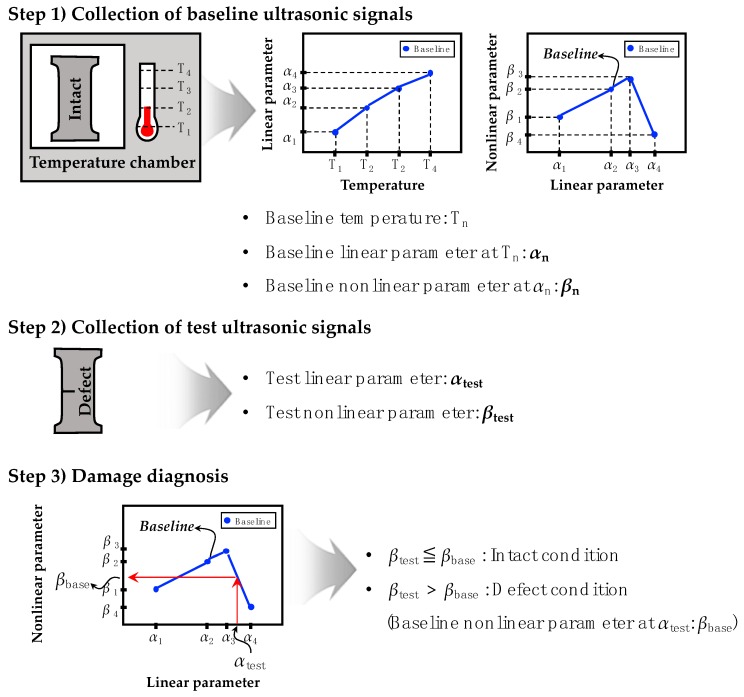
The schematic flow of the proposed damage diagnosis technique.

If the target structure is a linear system, $A_{2}$ will be zero, so β is also zero. However, no ideal linear system exists in practice. Sensors, target material, cables, and data acquisition system can all act as complex nonlinear sources, with inherent systematic nonlinearities. Although some inherent systematic nonlinearity is inevitable, a fatigue crack will act as the strongest nonlinear source thanks to the high sensitivity of super-harmonics to the fatigue crack. If a crack is initiated, β will increase remarkably. On the other hand, α will not be affected by the crack. One more interesting feature is that both α and β are sensitive to changes in temperature, but only β is sensitive to the fatigue crack. These unique characteristics of α and β are very promising for instantaneous and autonomous damage diagnosis that does not require a distinction between inherent systematic and crack-induced nonlinearities. Figure 1 illustrates the crack diagnosis procedure.

Step 1: Collection of baseline ultrasonic signals

First, multiple baseline data sets of ultrasonic wave signals are recorded from the intact structure under a range of temperatures, using a single embedded PZT. To minimize false alarms, a wide range of temperature conditions must be tested with small increments in this first step. Next, *α* and *β* are computed from the measured ultrasonic signals using Equations (8) and (9), respectively. Subsequently, an *α–β* baseline can be constructed by linear curve fitting to the *α* and *β* values.

Step 2: Collection of test ultrasonic signals

When an incipient fatigue crack is suspected in the target structure, a single set of test ultrasonic signals is collected in a similar manner as the previous step. In this case, the test temperature does not need to be recorded, because *α* is assumed to be proportional to the temperature change whether or not a fatigue crack is present. Hence, *α* can be used to compensate for undesired changes induced by changing temperature. Subsequently, *α_test_* and *β_test_* are computed using Equations (8) and (9), respectively. Note that the test data set should be obtained within the prescribed baseline data measurement condition ranges to avoid false alarms.

Step 3: Damage diagnosis

Once *α*_test_ is calculated in the previous step, the corresponding *β*_base_ value is automatically selected on the baseline and considered as the baseline nonlinear parameter. Physically, this selection process finds a set of baseline data, which is expected to be obtained from environmental conditions closest to the environment when the test data set is measured. Subsequently, damage diagnosis is performed based on the following criterion:

“If the test β data (β_test_) exceed the baseline nonlinear parameter (β_base_), a fatigue crack is detected. Otherwise, no crack alarm is triggered.”

This damage diagnosis criterion assumes that crack-induced nonlinearities will always be larger than inherent systematic nonlinearity and measurement errors. Again, this autonomous and instantaneous damage diagnosis enhances the applicability to real structures without any users’ intervention.

## 3. Finite Element Analysis

### 3.1. Description of Finite Element Models

**Figure 2:**
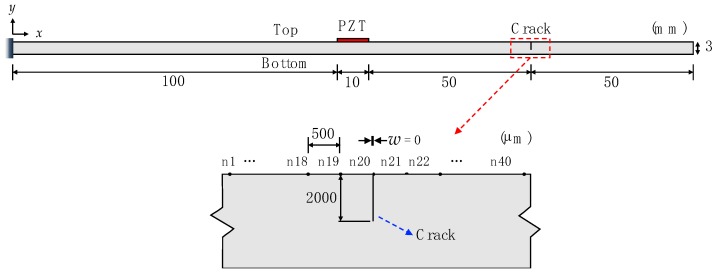
Schematic diagrams of the two-dimensional finite element model for the cracked condition with a zoomed-in view of the crack. PZT: piezoelectric transducer.

To investigate the existence and temperature dependency of the ultrasonic nonlinearity, 2D plain-strain models are prepared with and without a crack are made using four-node bilinear quadrilateral (CPE4R) elements and four-node bilinear piezoelectric plane stress (CPS4E) elements based on Abaqus 6.14 (Dassault Systems located in Johnston, Rhode Island, United States) [21]. The 2D models of an aluminum cantilever beam have dimensions of 210 × 3 mm, and APC 850-type PZTs (American Piezo Ceramics International located in Mackeyville, Pennsylvania, United States) [22] with a dimension of 10 × 0.508 mm are attached to the top surface of the beams as shown in Figure 2. The material properties of the aluminum beams and PZTs used in this simulation are summarized in Table 1 and Table 2, respectively. In case of the crack model, a closed crack is modeled as the surface-to-surface contact condition shown in Figure 2. The crack depth is 2 mm, and it is located 55 mm away from the center of the PZT.

The PZT attached to the top surface is used to generate Lamb waves at the ultrasonic frequency of 100 kHz by applying the input waveform of a tone burst. Then, the reflected nonlinear ultrasonic waves are recorded at a sampling rate of 20 MHz. The spatial and time resolutions should be well designed to generate an accurate simulation. The mesh size is 500 μm, and a total of 2972 nodes and 2540 elements are used in each simulation. Using the geometric and material properties defined above, simulations were performed for the temperature range of −30 °C to 60 °C at increments of 10 °C (10 cases), using input voltages ranging from 12 to 36 V at 6 V increments (5 cases). Thus, a total of 100 simulation cases are computed for intact (50 cases) and cracked models (50 cases).

### 3.2. Finite Element Analysis Results

**Figure 3:**
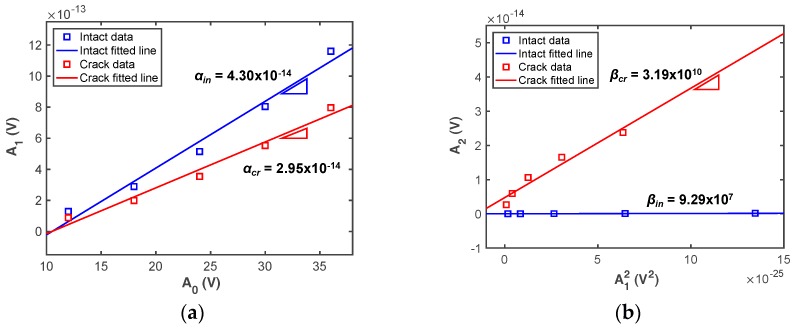
Comparison of representative simulation results between intact and cracked conditions at 10 °C: (**a**) *α*, (**b**) *β*. Subscripts in and cr denote intact and cracked conditions, respectively.

Based on the FE analysis results, *α* and *β* are calculated using a linear curve-fitting method. The gradient relations between *A*_0_ vs. *A*_1_ and *A*_1_^2^ vs. *A*_2_ can be computed using Equations (8) and (9) when the applied voltages increase. Figure 3 shows representative FEM results of the intact and cracked cases obtained at 10 °C. No significant difference between the *α* values obtained from the intact and cracked FE models appears, whereas the *β* values are remarkably altered by the fatigue crack as the applied voltage increases. This indicates that *β* is much more sensitive to fatigue cracks than *α*, as expected. In practice, the linear ultrasonic method is not useful for detecting fatigue cracks because a difference in *α* cannot be clearly observed between the intact and cracked cases [24]. However, a difference between intact and cracked *α* values can be observed in Figure 3a. This result can be explained if we notice that the simulated crack interface is much more distinct than a realistic rough crack surface, which leads to the partial generation of linear ultrasonic wave reflections within the crack interface [7].

**Figure 4:**
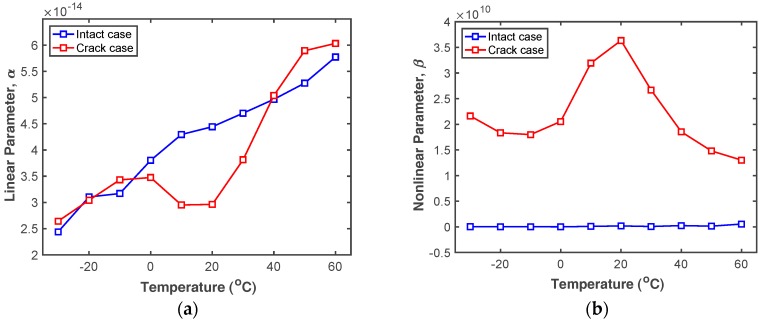
Comparison of (**a**) *α* and (**b**) *β* obtained from the intact and cracked FE models at different temperatures.

The trends in *α* and *β* over the 100 simulation cases are plotted in Figure 4. Figure 4a shows that *α* is almost linearly proportional to the temperature increment in the intact case. Although *α* of the cracked case fluctuates as temperature changes, the trend is similar to the intact case in terms of unit order. By contrast, the trends of the intact and cracked *β* values are totally different, as can be seen in Figure 4b. This indicates that *β* is sensitive to both temperature changes and the presence of a fatigue crack.

In summary, the simulation results of Figure 3 and Figure 4 indicate that (i) *α* is not affected significantly by the presence of a fatigue crack, but responds almost linearly to temperature; (ii) *β* is dramatically changed by presence of a fatigue crack under certain temperature conditions; (iii) the fatigue crack may not be effectively identified by *β* alone, since *β* is also affected by changes in temperature. Therefore, the simultaneous use of *α* and *β* is promising for fatigue crack detection under varying temperature.

## 4. Experimental Validation

### 4.1. Description of Experimental Setup

**Figure 5:**
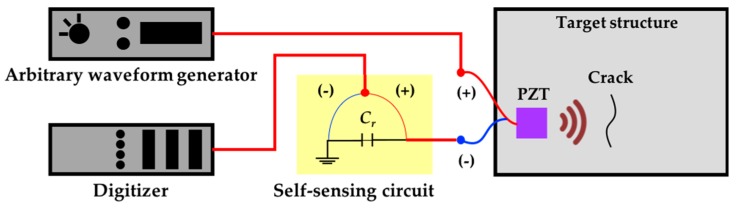
Schematic design of the self-sensing nonlinear ultrasonic system.

To experimentally validate the proposed technique, a self-sensing nonlinear ultrasonic system was prepared as diagrammed in Figure 5. A single PZT is used to simultaneously generate and measure nonlinear ultrasonic waves. The use of only a single PZT minimizes the implementation problems associated with complicated installation and electrical distortions caused by electrical equipment [16,17]. A 16-bit arbitrary waveform generator (AWG) is used to generate the ultrasonic signal and is connected to the PZT directly. A self-sensing circuit [25,26] is inserted between the PZT and a 14-bit digitizer (DIG) to detect ultrasonic nonlinearities, as shown in Figure 5.

**Figure 6:**
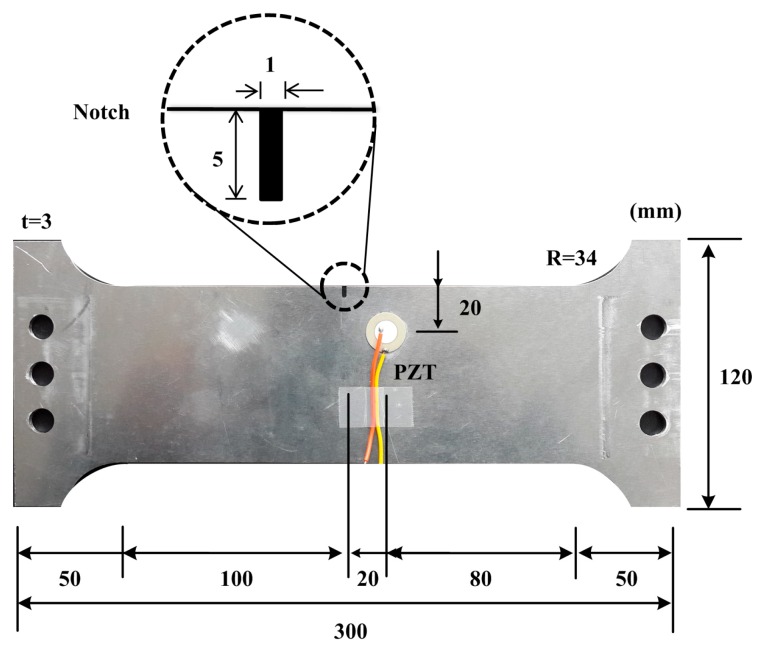
Target structure with the self-sensing nonlinear ultrasonic system installed.

**Figure 7:**
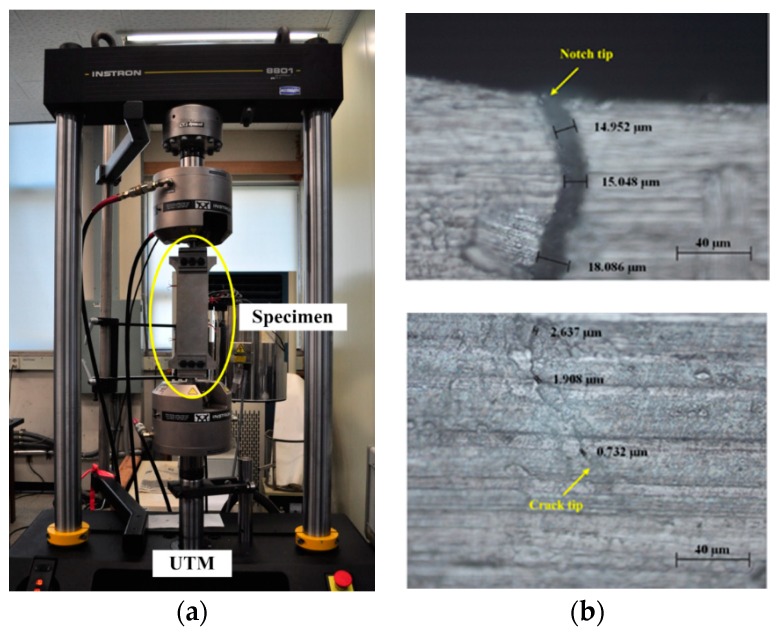
Creation of a fatigue crack through cyclic loading tests: (**a**) Tensile-loading test setup with a specimen and (**b**) Microscopic images and crack widths. UTM: universal testing machine.

To experimentally investigate the existence and temperature dependency of the ultrasonic nonlinearity, an aluminum dog-bone shape specimen was prepared as shown in Figure 6. The dimensions of the specimen are 300 × 120 × 3 mm. An initial notch of 1 × 5 mm is introduced at the top-center of the specimen to concentrate stress and encourage the formation of a fatigue crack. Using a universal testing machine as shown in Figure 7a, the fatigue crack is then created by applying cyclic tensile loads ranging from 1.6 kN to 16 kN with a loading cycle of 10 Hz. This loading scheme produces a 13 mm-long fatigue crack from the notch tip after 130,000 loading cycles. Microscope images reveal that the fatigue crack widths are approximately 16.03 μm around the notch tip and 1.76 μm at the crack tip, as shown in Figure 7b. An APC 850-type PZT with a diameter of 20 mm and a thickness of 0.508 mm is attached on the surface of the beam, and is placed 20 mm away from both the upper edge of the specimen and the center of the crack, as shown in Figure 6.

**Figure 8:**
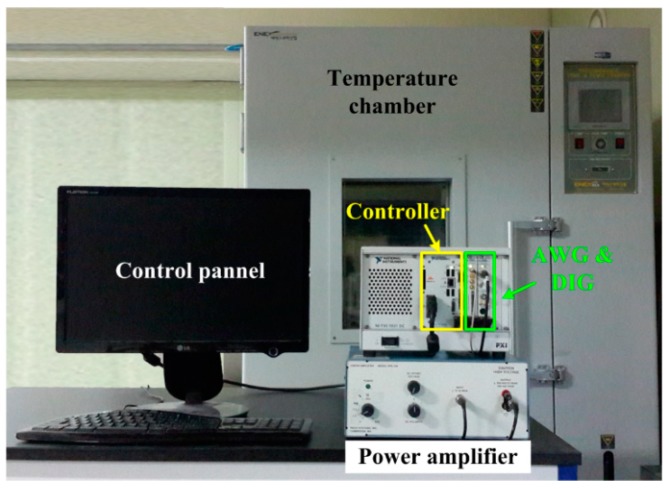
Self-sensing nonlinear ultrasonic system for temperature variation tests. AWG: arbitrary waveform generator; DIG: 14-bit digitizer.

Once the fatigue crack was created, detection tests were carried out using the self-sensing nonlinear ultrasonic system, as shown in Figure 8. The system includes a controller, an AWG, a DIG, a power amplifier, and a temperature chamber for temperature variation tests. Once the controller sends out control signals to the AWG and DIG, the AWG activates the PZT to generate ultrasonic waves with a chirp waveform. Simultaneously, the DIG gathers corresponding responses from the same PZT through the self-sensing circuit. Then, the measured data are transmitted to the controller and stored for signal processing. In these tests, the chirp waveform had frequencies ranging from 100 kHz to 120 kHz, and the input voltages are gradually increased from 12 Vpp to 36 Vpp with an increment of 6 Vpp. The sampling frequency was 5 MHz, and steady-state responses are measured over 0.2 s. Each response is measured five times in the time domain, and these samples are averaged to improve the signal-to-noise ratio.

The temperature-variation tests used the following steps. First, baseline data are obtained from the intact condition of the target specimen under the temperature range of −10 °C to 40 °C with the increment of 10 °C. Then, another data set of the intact condition was obtained at the temperatures of −3 °C, 15 °C, and 22 °C to perform false-positive tests. These temperatures do not overlap with those in the baseline tests. Next, a crack is created in the target specimen, and false-negative tests are performed with the cracked specimen under the temperatures of 0 °C, 7 °C, 18 °C, 30 °C and 35 °C. These test temperatures were randomly selected. The test scenarios under varying temperatures are summarized in Table 3.

### 4.2. Experimental Results

#### 4.2.1. Frequency Domain Results

**Figure 9:**
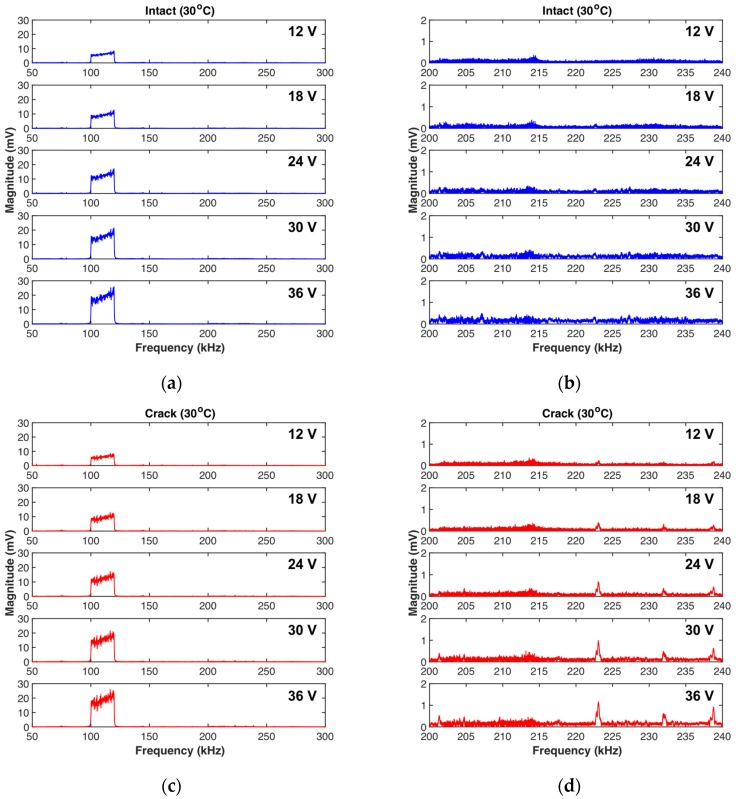
Representative test results in the frequency domain: (**a**) Primary and (**b**) 2nd harmonic responses of the intact case, and (**c**) primary and (**b**) 2nd harmonic responses of the cracked case at 30 °C.

Figure 9 shows representative test results in the frequency domain obtained at 30 °C. The primary frequency components are not significantly changed by a fatigue crack, as observed in Figure 9a,c. On the other hand, the 2nd harmonic responses are affected by the fatigue crack, as comparison of Figure 9b with Figure 9d shows. In particular, the responses at several specific frequencies are most sensitive to the fatigue crack; these frequencies are 223 kHz and 232 kHz in this case. This finding reveals that the chirp input waveform designed with a wide frequency range is more effective for detecting fatigue cracks.

#### 4.2.2. Estimation of Linear and Nonlinear Parameters

**Figure 10:**
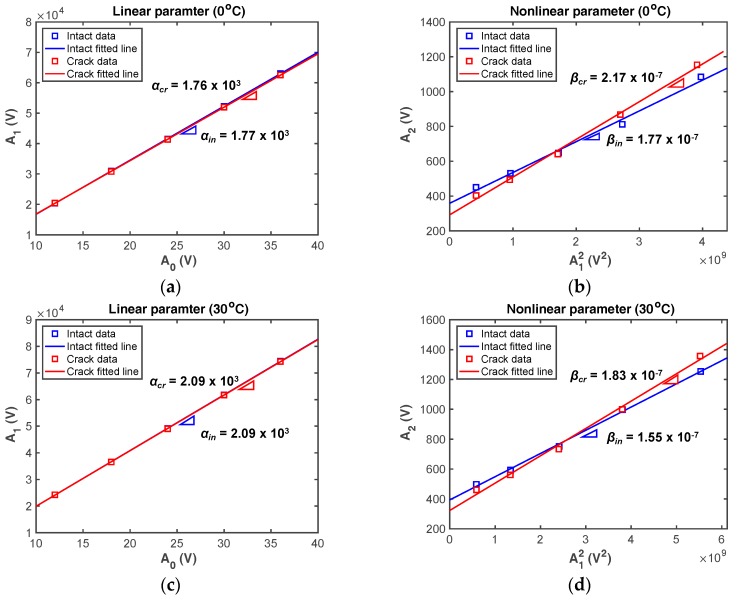
Comparison of representative experimental results between intact and cracked samples tested at 0 °C and 30 °C: (**a**,**c**) *α*, (**b**,**d**) *β*. The subscripts **in** and **cr** denote intact and cracked conditions, respectively.

Based on the frequency-domain results, *α* and *β* are calculated from the primary frequency range from 100 kHz to 120 kHz and their double frequency range from 200 kHz to 240 kHz, as the applied voltages increase. Figure 10 shows representative calculation results obtained at 0 °C and 30 °C. As expected, there is no remarkable difference between the *α* values obtained from the intact and cracked cases. As mentioned in Section 3.2, the *α* values of the experimental intact and cracked cases shown in Figure 10a,c are more like each other than are the simulated results, because the rough and nearly closed crack interface does not affect *α* in the experimental case [7]. This means that *α* is not suitable for the detection of fatigue cracks, especially closed cracks. However, *β* is affected by the existence of a fatigue crack, as shown in Figure 10b,d, revealing that it is possible to identify a fatigue crack using *β*. Note that *β* obtained from the intact condition should theoretically be zero, but it is not due to inherent systematic nonlinearities and measurement errors.

**Figure 11:**
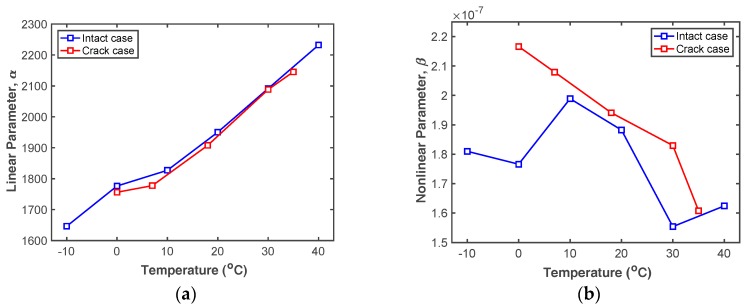
Comparison of (**a**) *α* and (**b**) *β* obtained from the intact and cracked specimens under changing temperature.

Once *α* and *β* values are computed for all test cases of interest, their tendencies can be plotted with varying temperature as shown in Figure 11. The trends of the computed *α* and *β* values fluctuate similarly to the simulated results shown in Figure 4, but the trends are not the same due to the inherent systematic nonlinearities and the measurement errors as mentioned above. Figure 11a shows that *α* obtained from both the intact and cracked specimens are almost linearly proportional to the temperature and similar to each other. Thus, fatigue cracks cannot be detected by *α* alone, as expected. *β* is sensitive to temperature variation in both cases, but the *β* obtained from the cracked specimen is always greater than that from the intact specimen, as shown in Figure 11b. Thus, fatigue cracks can be detected by measuring *β* and considering the test temperature.

#### 4.2.3. Decision-Making

**Figure 12:**
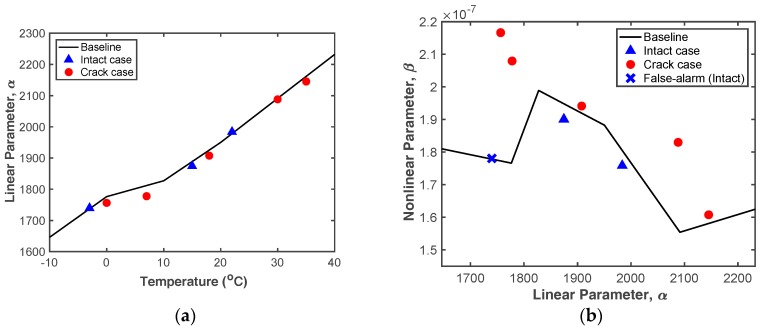
Experimental results: (**a**) Temperature vs. *α*, (**b**) *α* vs. *β*.

Figure 12 shows the validation test results computed by the proposed crack diagnosis procedure. As expected, it is difficult to distinguish intact and cracked cases using only the *α* parameter, since *α* is not significantly affected by the existence of a fatigue crack but is sensitive to temperature variation, as shown in Figure 12a. In contrast, the crack-test data clearly exceed the baseline, with only one false alarm appearing in Figure 12b. Here, the false alarm case exceeds the baseline, having an error of 0.12% compared with the baseline. The existence of this false alarm shows that the *α–β* curve is not simply a straight line in the temperature range of −10 °C to 0 °C, but fluctuates due to inherent systematic nonlinearity and measurement error. In the FEM simulation, it is similarly seen that the *α–β* curve is remarkably affected when the test temperature is in the range of −10 °C to 40 °C. To minimize false positives, a wide range of temperature conditions must be tested with a small increment when intact data sets are collected.

Furthermore, the cracked *β* is always considerably larger than the baseline, meaning that the fatigue crack-induced *β* is much larger than any systematic nonlinearity. This indicates that *β* is an effective indicator of fatigue cracks.

## 5. Discussion

The proposed crack detection technique represents a promising alternative to existing methods. However, it should be noted that the crack detection technique is specific to the test specimens and the PZT configurations used in this study, and caution is advised before generalizing the findings presented here to other applications. For example, the performance of the proposed technique can be changed by the temperature increment, the target material’s homogeneity, crack length, crack type, the distance between crack and PZT and so on. As the follow-up work, this device will be, therefore, tested for the detection of various damage types and temperature increment under realistic use conditions. In addition, the sensor’s applicability to structures with more-complex boundary conditions will be addressed.

## 6. Conclusions

This paper describes a novel nonlinear ultrasonic fatigue crack detection technique that can be applied in conditions of varying temperature and requires only a single PZT. The linear parameter *α* is used to compensate for systematic nonlinearity and the effects of temperature, and the nonlinear parameter *β* is used to identify fatigue cracks. Since a single PZT is used to both generate waveforms and sample ultrasonic nonlinearities, the system is relatively simple and cost-effective. Moreover, this system can be used to minimize electrical distortions related to the data acquisition system, sensors, and electric wires, which are a critical source of noise in such applications.

FEM simulations were used to confirm the pattern of the change in nonlinear response under conditions with varying temperature. Then, the technique was experimentally validated in tests with a fatigued aluminum plate in an oven of varying temperature. These validation results indicate that the parameter α is not affected by the presence of a fatigue crack but does change linearly with temperature. On the other hand, *β* is sensitive to temperature and the presence of a fatigue crack. Therefore, measuring both *α* and *β* simultaneously offers a unique solution for detecting fatigue cracks in an environment with temperature variations.

## Figures and Tables

**Table 1:** Material properties of the aluminum cantilever beam model at various temperatures.

Temperature (°C)	Density (kg/m^3^)	Young’s Modulus (GPa)
−100	2791.0	78.2
−75	2785.5	77.3
−50	2780.0	74.3
−25	2774.5	73.3
0	2769.0	72.4
25	2763.5	68.0
50	2758.0	67.1
75	2752.5	66.1
100	2747.0	65.7

**Table 2:** Material properties of the PZT at various temperatures [23].

Temperature (°C)	Dielectric Const. (*x*-axis)	Dielectric Const. (*y*-axis)	Piezoelectric Coef. (d15) (m/V)	Piezoelectric Coef. (-d31) (m/V)	Piezoelectric Coef. (d33) (m/V)
−100	8.4 × 10^−9^	7.3 × 10^−9^	6.9 × 10^−10^	−1.6 × 10^−10^	4.4 × 10^−10^
−75	9.7 × 10^−9^	8.4 × 10^−9^	7.0 × 10^−10^	−1.8 × 10^−10^	4.7 × 10^−10^
−50	11.1 × 10^−9^	9.6 × 10^−9^	7.1 × 10^−10^	−2.1 × 10^−10^	5.0 × 10^−10^
−25	12.4 × 10^−9^	10.7 × 10^−9^	7.2 × 10^−10^	−2.3 × 10^−10^	5.3 × 10^−10^
0	13.7 × 10^−9^	11.9 × 10^−9^	7.3 × 10^−10^	−2.5 × 10^−10^	5.6 × 10^−10^
25	15.1 × 10^−9^	13.0 × 10^−9^	7.4 × 10^−10^	−2.7 × 10^−10^	5.9 × 10^−10^
50	16.4 × 10^−9^	14.2 × 10^−9^	7.5 × 10^−10^	−3.0 × 10^−10^	6.3 × 10^−10^
75	17.7 × 10^−9^	15.3 × 10^−9^	7.6 × 10^−10^	−3.2 × 10^−10^	6.6 × 10^−10^
100	19.0 × 10^−9^	16.5 × 10^−9^	7.7 × 10^−10^	−3.4 × 10^−10^	6.9 × 10^−10^

**Table 3:** Test scenarios under temperature variation.

Data	Temperature (°C)	Condition
Baseline	−10, 0, 10, 20, 30, 40	Intact
False-positive test	−3, 15, 22	Intact
False-negative test	0, 7, 18, 30, 35	Cracked
